# Simultaneous recovery of high-purity copper and polyvinyl chloride from thin electric cables by plasticizer extraction and ball milling†

**DOI:** 10.1039/c8ra00301g

**Published:** 2018-02-13

**Authors:** Jing Xu, Naoki Tazawa, Shogo Kumagai, Tomohito Kameda, Yuko Saito, Toshiaki Yoshioka

**Affiliations:** Graduate School of Environmental Studies, Tohoku University 6-6-07 Aoba Aramaki-Aza, Aoba-ku Sendai Miyagi 980-8579 Japan kumagai@env.che.tohoku.ac.jp

## Abstract

Herein, we introduce a combination of plasticizer extraction from polyvinyl chloride (PVC) and ball milling for the simultaneous, effective recovery of PVC and copper (Cu) from thin electric cables. PVC coverings typically contain plasticizers for flexibility. As such, PVC cables become brittle after plasticizer extraction, causing them to be easily crushed by physical impact. Hence, we extracted the plasticizers from the PVC coverings of electric cables using organic solvents, and then crushed the obtained cable samples by ball milling. The influences of the plasticizer extraction yield and PVC morphologies before and after extraction on separation by ball milling were investigated. After a series of treatments to PVC coverings including quantitatively de-plasticizing for 5 h by Soxhlet-extraction in diethyl ether, 6 h ball milling and 1 h shaking in the sieve shaker, a maximum separation rate of 77% was achieved and the purity of the obtained separated Cu reached >99.8%.

## Abstract 2

Herein, we introduce a combination of plasticizer extraction from polyvinyl chloride (PVC) and ball milling for the simultaneous, effective recovery of PVC and copper (Cu) from thin electric cables.

## Graphical abstract

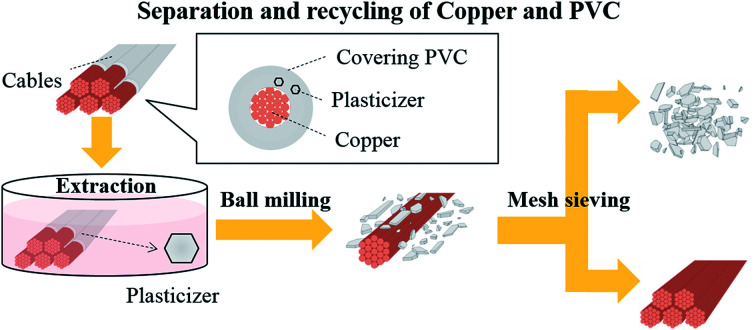

## 1. Introduction

Polyvinyl chloride (PVC) has become an indispensable resin because of its high chemical and physical stability. Compared with other resins, PVC consists of a high percentage of Cl (57 wt%), which allows its combination with a wide variety of additives such as plasticizers, stabilizers, flame retardants, and fillers. As a result, PVC is used in a wide variety of fields including the construction, automobile, electrical, medical, and daily goods industries.^1^ The global production of PVC reached 41 420 kt in 2015,^2^ making it the third highest in volume among plastic resins.

Electric cables typically consist of Cu covered with PVC, and their use represented 3080 kt of global PVC demand in 2013.^3^ Cu is well known to be an expensive and in-demand metal, while well-separated PVC covering may be mechanically recycled into other PVC products. Therefore, there is a huge need for effective techniques to separate used electric cables into Cu and PVC with high purity.

Electric cables are roughly divided into thick (cm-order diameter) and thin (mm-order diameter) cables; thick cables are used for electric power transmission lines, while thin cables are used for wire harnesses in automobiles as well as electrical and electronic equipment (EEE).^4^ Globally, the demand for automobiles^5–7^ and EEE^8,9^ has been significantly increasing every year; the demand for thin cables therefore is also expected to increase in the future. Thick cables can be easily separated into high-purity PVC and Cu by peeling or shredding techniques.^10^ Current peeling equipment, however, cannot handle thin cables, and shredding techniques produce fine particles, which result in the low-purity separation of both the Cu and PVC.^11^ For the above reasons, in the case of Japan, almost all unsupported thin cables are exported to developing countries. This situation is likely similar in other advanced countries. Generally, these exported cables are either manually peeled by low-wage workers, or only the Cu is recovered while the PVC covering is burned. These techniques are not only ineffective, but also cause air, soil, and groundwater pollution if open burning or dumping is carried out.^12–14^ In the case of automobiles^15,16^ and waste EEE (WEEE),^17,18^ parts of the wire harnesses are shredded with other components and then disposed of by incineration or landfill.

Thermal treatment resulting in energy recovery, such as pyrolysis, gasification, and incineration, in which PVC is used as a hydrocarbon source, has been widely reported for PVC-containing waste.^19^ However, a significant problem is the handling of the chlorine in the PVC. Fonseca *et al.*^20^ reported that the dehydrochlorination and hydrolysis of plasticizers occurred during the thermal treatment of flexible PVC with steam. In addition, Zablocka-Malicka *et al.*^21^ and Szczepaniak *et al.*^22^ investigated the steam gasification of PVC multi-wire constructs, which resulted in the Cu not undergoing any changes as a result of the treatment. However, thermal treatments suffer from the following drawbacks: extra energy for heating is necessary, and acidic hydrogen chloride (HCl) gas is emitted, which has to be captured by absorbents such as Ca(OH)_2_ to prevent damage to the industrial equipment.^23^ If the temperature is relatively low, there is the additional risk of producing chlorinated organics such as toxic dioxins.^24^

Several studies have reported on laboratory-scale processes to overcome these obstacles associated with thin electric cables. For example, Sheih and Tsai^25^ reported on the perfect recovery of both Cu and PVC using a hot water separation process. In brief, wires were cut into 3 to 5 mm long pieces (bigger than the pieces in the common shredding process), which were then mixed with hot water at 80 °C in a blender at 4200 rpm for 5 min, resulting in the swelling of the PVC and its separation from the Cu. The PVC and Cu could then be separately recovered by heavy liquid separation using CCl_4_ and C_2_H_2_Br. Liu *et al.*^26^ employed an ultrasonic technique to produce cavitation between the PVC covering and Cu. This technique also achieved the perfect recovery of both the Cu and PVC using electric cables 1–2 mm in length and only required the water to be heated to 60 °C. Liu *et al.*^27^ patented a technology using ultrasonic to separating waste cables by which the waste cables in 3–5 mm were frozen at −10 °C and immersed in the ultrasonic for 0.5–2 h at 40–60 °C, then treated by ultrasonic at 40–50 °C, an effective separation of non-metal and metal more than 90% was achieved. Although the three methods are attractive for achieving a high separation accuracy, both require heating and have only been applied to small sample sizes. In addition, a freezing technology reported by You *et al.*^28^ for recycling waste wires. The wires in small size were frozen by liquid nitrogen for 10 min to make the plastic coverings brittle, which was easy to be crushed and separated by oscillation screen. This method got high recovery and no waste liquid and gas generated, however, the high demands on the freezing equipment made it expensive. Recently, Park *et al.*^29^ and Dascalescu *et al.*^30^ published electrostatic separation of crushed waste cables in 1–4 mm, which improved the low efficiency of gravity separation and avoided using toxic organic solvents but the loss of copper was inevitable, therefore, the more convenient and precise methods should be developed.

**Fig. 1:**
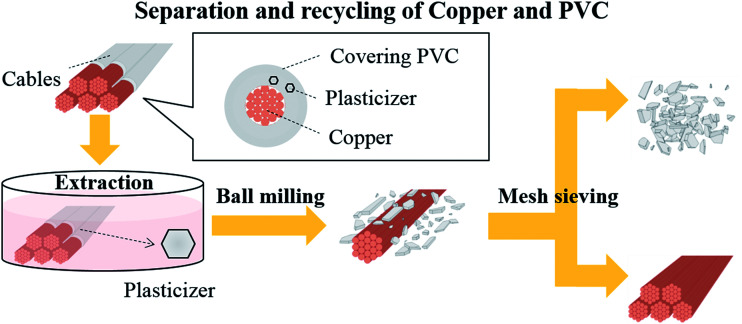
Schematic representation of the separation and recycling of copper and polyvinyl chloride (PVC) from electric cables.

In this work, we have developed a novel method that combines plasticizer extraction and ball milling for the recycling of thin electric cables (Fig. 1). The advantages of this technique include its compatibility with longer thin cables (1 cm in length) and its energy consumption being significantly lower than that of conventional thermal processes. In addition, there is no need for special equipment that can resist heat and high pressures, as well as ultrasonic generators. All the PVC coverings contain plasticizers such as dioctyl phthalate (DOP) and diisononyl phthalate (DINP) to ensure flexibility, which are physically kneaded into the PVC matrix. Therefore, in the new process, plasticizer extraction from the cables is first carried out at a moderate temperature to yield a “brittle” PVC covering, with the extracted plasticizers collected in their original form. Then, the hardened electric cables are subjected to ball milling, which crushes only the PVC covering and removes it from the Cu wires. Finally, the PVC covering and Cu are separated by simple mesh sieving.

In the present work, thin commercial electric cables (2.1 mm in diameter) containing DINP were treated with several organic solvents. The effects of each solvent type on the plasticizer extraction yield and the morphology changes of the PVC covering during the extraction procedure were investigated. Finally, the effects of the extraction conditions on the separation rate and purity of the recovered Cu and PVC during ball milling were evaluated.

## 2. Experimental section

### 2.1 Materials

Commercial electric cables (KV-0.75-W-100, 2.1 mm in diameter) were purchased from MISUMI Group Inc. (Tokyo, Japan) and cut into pieces 1 cm in length. In total, 120 cables (equal to 12 g) were used for all experiments. The cable samples consisted of 63 wt% Cu wire and 37 wt% PVC. The elemental composition of the PVC covering was as follows: 35.8% C, 4.5% H, 0.2% N, 25.2% Cl, and 34.3% other components (balance), which includes oxygen and ash. The plasticizer content was identified and quantified by Soxhlet extraction using diethyl ether and ^1^H nuclear magnetic resonance analysis (^1^H-NMR) by an Avance 400 MHz (Bruker, Rheinstetten, Germany). The conditions used for the extraction procedure and ^1^H-NMR analysis, as well as the obtained ^1^H-NMR spectra, are given in the ESI.† The PVC covering was found to contain 18 wt% DINP.

Diethyl ether, methanol, hexane, toluene, ethyl acetate, *N*,*N*-dimethylformamide, and ethanol were obtained from Kanto Chemical Co., Inc. (Tokyo, Japan). Finally, pure PVC resin was purchased from Wako Pure Chemical Industries, Ltd. (Osaka, Japan).

### 2.2 Solvent selection through swelling and solubility tests of PVC

To select a feasible extraction solvent, diethyl ether, methanol, hexane, toluene, ethyl acetate, *N*,*N*-dimethylformamide, and ethanol were examined as candidates. They all have comparatively low boiling points and are less toxic than chloroform^31^ or dichloromethane,^32^ which is advantageous for extraction. The boiling point, Hildebrand solubility parameter (SP),^33^ and Hansen solubility parameter (HSP)^34^ values for all these solvents are summarized in Table 1 in the results section, together with the parameters reported for PVC and DINP for comparison.^35^

**Table 1:** Properties of materials and organic solvents. HSP: Hansen solubility parameter; DINP: diisononyl phthalate; *R*_swell_: swelling rate; *δ*_SP_: Hildebrand solubility parameter

Materials	B.P.^a^ [°C]	*δ*_SP_ [MPa^1/2^]	HSP				*R*_swell_ [−]
			*δ*_d_	*δ*_p_	*δ*_h_	*δ*_HSP_ = (*δ*_d_^2^ + *δ*_p_^2^ + *δ*_h_^2^)^1/2^	
PVC	—b	9.5	18.8	9.2	6.3	21.9	—
DINP	405.7	8.5	16.6	6.6	2.9	18.1	—
Hexane	68.7	7.3	14.9	0.0	0.0	14.9	1.1
Diethyl ether	34.6	7.4	14.5	2.9	4.6	15.5	1.2
Toluene	110.6	8.9	18.0	1.4	2.0	18.2	1.5
Ethyl acetate	77.1	9.1	15.8	5.3	7.2	18.1	2.5
*N*,*N*-Dimethylformamide	152.8	—b	17.4	13.7	11.3	24.9	Dissolution
Ethanol	78.4	12.7	15.8	8.8	19.4	26.5	1.0
Methanol	64.5	14.5	14.7	12.3	22.3	29.4	1.0

a Boiling point.

b Not reported.

The SP value is a numerical value that comes from the cohesive energy density of a solvent and indicates its dissolution capacity.^36^ The SP (*δ*_SP_ [MPa^1/2^]) can be expressed by eqn (1):1
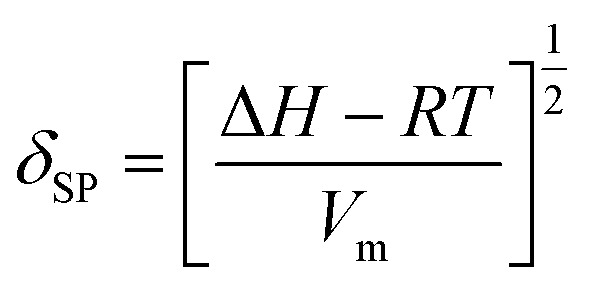
where Δ*H* is the heat of vaporization, *V*_m_ [m^3^ mol^−1^] is the molar volume, *R* [J (mol^−1^ K^−1^)] is the gas constant, and *T* [K] is the absolute gas temperature.

The HSP is well known for its wide applicability in the prediction of the compatibility of two combined materials, since materials with similar HSP values exhibit a physical affinity to one another.^37^ Unlike the SP, the HSP concept relates to the cohesive energy of a liquid, which can be divided into three types of interactions: the dispersion (d), polar (p), and hydrogen bonding (h) components. These three interaction types are described by the Hansen parameters *δ*_d_, *δ*_p_, *δ*_h_, respectively. The total HSP (*δ*_HSP_ [MPa^1/2^]) can then be expressed by eqn (2):2
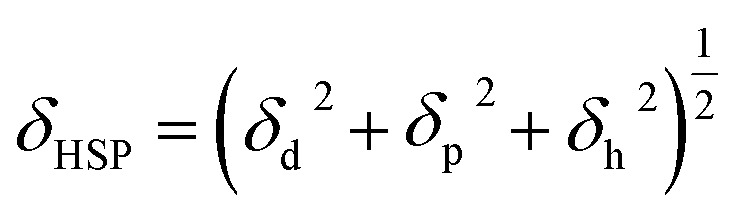


The swelling rate (*R*_swell_) and solubility of the PVC resin in each solvent were investigated. In short, electric cables, 8 cm in length, were submerged in 100 mL of organic solvent in a sealed 110 mL bottle at room temperature for 60 min. The volume of the cables before and after being submerged were measured by the electronic densimeter (MDS-300, Alfa Mirage Co., Ltd., Osaka, Japan).^38^ Based on this method, the *R*_swell_ [−] is defined by eqn (3):3
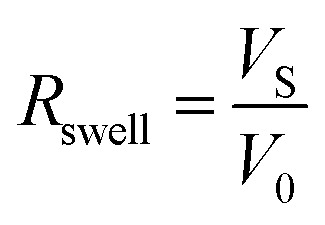
where *V*_S_ and *V*_0_ are the volumes of the cables after and before being submerged, respectively, as well as solubility and other properties of each solvent, the three most feasible solvents were selected. These were diethyl ether, methanol, and hexane.

### 2.3 Plasticizer extraction

The extraction of the DINP from the cable samples was carried out by Soxhlet extraction. Briefly, cables (12 g) were placed into a Soxhlet extractor filled with 150 mL of diethyl ether, methanol or hexane. Diethyl ether was heated at 75 °C and both methanol and hexane were heated at 120 °C, which were about twice as much as their boiling points. These temperatures were chosen to attain a higher refluxing rate at about 3 drops per s in the extractor, which was known to be a suitable rate for Soxhlet extraction.^39^ The Soxhlet extraction was carried out for a maximum of 300 min. To obtain a low control extraction yield (*Y*_extraction_), the same amount of cables was submerged in diethyl ether at ambient temperature for a maximum period of 300 min. The *Y*_extraction_ [%] value was then calculated using eqn (4):4
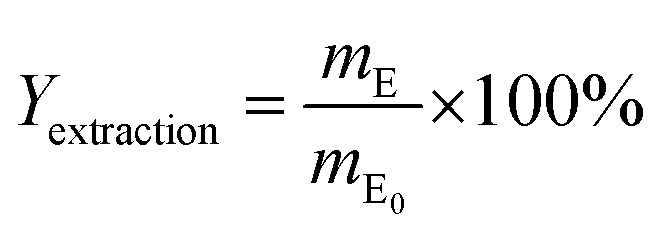
where *m*_E_0__ [g] is the original amount of DINP in the sample and *m*_E_ [g] is the mass of extracted DINP, defined by eqn (5) and (6), respectively:

5 *m*_E_0__ = *w*_0_ × 0.37 × 0.18

6 *m*_E_ = *w*_0_ − *w*_E_

where *w*_0_ [g] is the mass of the cable sample before extraction and *w*_E_ [g] is the mass of the cable samples after extraction and overnight drying in a vacuum at 40 °C.

The morphologies of the samples before and after extraction were analyzed using scanning electron microscopy (SEM) (TM-3000, Hitachi, Ltd., Tokyo, Japan), while their crush resistances were determined using a compression testing machine (AG-X 50 kN, Japan Electric Cable Technology Center, Shizuoka, Japan).

### 2.4 Shrinking test

To measure the shrinking rate (*R*_shrink_) of the electric cables, they were cut into pieces with a length (*L*_0_) of 20 cm and submerged in 100 mL of organic solvent (diethyl ether, hexane, or methanol) for different lengths of time. After submerging and drying, the length of each cable sample was measured with a ruler and the *R*_shrink_ [%] value calculated using eqn (7):7
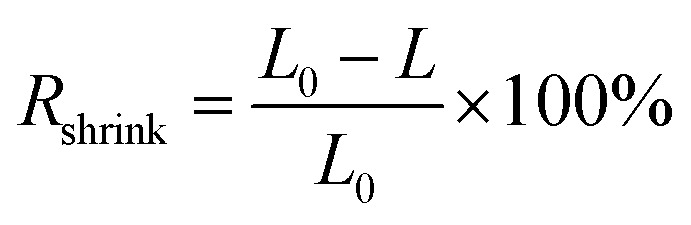
where *L* [cm] is the length of the cable sample after drying.

### 2.5 Ball milling

Ball milling tests on the untreated and plasticizer-extracted samples with different *Y*_extraction_ values were carried out using a stainless-steel ball mill reactor (diameter: 15 cm; PM-001, AS ONE Co., Tokyo, Japan) with 20 tungsten carbide balls (diameter: 1 cm; weight: 7.8 g per ball; AS ONE Co.) at 45 rpm for 6 h. To determine the size distribution of the crushed samples, they were further separated into 12 samples ranging in size between 100 μm and 4.75 mm using an electromagnetic sieve shaker (vibrations: 3000 per min; amplitude: 1.8 mm; AS200Basic, AS ONE Co.) for 1 h. The separation rate (*R*_separation_ [%]) of each cable sample was defined by eqn (8):8
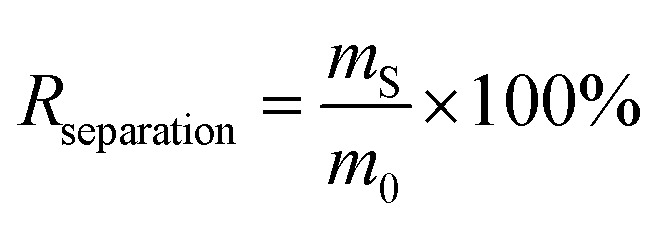
where *m*_S_ [g] is the amount of separated cable. The Cu purity (*P*_Cu_ [%]) was defined by eqn (9):9
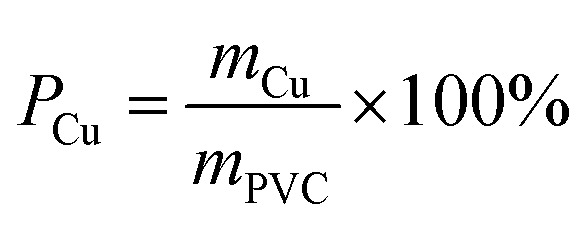
where *m*_PVC_ [g] and *m*_Cu_ [g] are the amounts of PVC and Cu, respectively, in the sieved fraction from 100 to 600 μm. The latter arose because all the separated Cu was confined to the size range between 100 and 600 μm. This fraction is therefore the only one for which the weight of the PVC was necessary. For those samples with sizes of more than 600 μm, only separated PVC or unseparated cables remained.

## 3. Results and discussion

### 3.1 Swelling and solubility test

The *R*_swell_ values for PVC obtained for different solvents are summarized in Table 1. The solvent *N*,*N*-dimethylformamide could dissolve the PVC completely, rather than causing it to swell, while ethyl acetate, toluene, diethyl ether and hexane caused the PVC to swell to different degrees. No significant changes were observed for ethanol and methanol. Among the *δ*_SP_ values listed in Table 1, ethanol (12.7) and methanol (14.5) differ the most from PVC (9.5), while the values of toluene (8.9) and ethyl acetate (9.1) are closest to PVC. And hexane (7.3) and diethyl ether (7.4) have appropriate values between the two kinds of solvents above. It showed that the ability of solvents to make PVC swelling is depending on the difference between the *δ*_SP_ values of solvents and PVC. Even though the *δ*_HSP_ of PVC (21.9), toluene (18.2), ethyl acetate (18.1), and *N*,*N*-dimethylformamide (24.9) are similar, the PVC did not dissolve in either the toluene or ethyl acetate due to the values of *δ*_d_, *δ*_p_, and *δ*_h_ being significantly different. The similar *δ*_d_ (17.4), *δ*_p_ (13.7), and *δ*_h_ (11.3) values of *N*,*N*-dimethylformamide, compared to those of PVC, on the other hand, resulted in complete PVC dissolution. This clearly suggests that a comparison of each separate component in terms of the *δ*_SP_ value (*δ*_d_, *δ*_p_, and *δ*_h_) enables a better prediction of the compatibility between the PVC, plasticizer, and organic solvent than *δ*_SP_ and *δ*_HSP_ alone. Since the purpose of our extraction process is plasticizer removal without any changes to the PVC resin to enable the latter's mechanical recycling, diethyl ether, hexane, and methanol were selected for the DINP extraction; these solvents do not cause the PVC to swell much and have comparatively low boiling points (which is good for the recovery of both the solvent and plasticizer).

### 3.2 Effects of solvent type on extraction yield of DINP

**Fig. 2:**
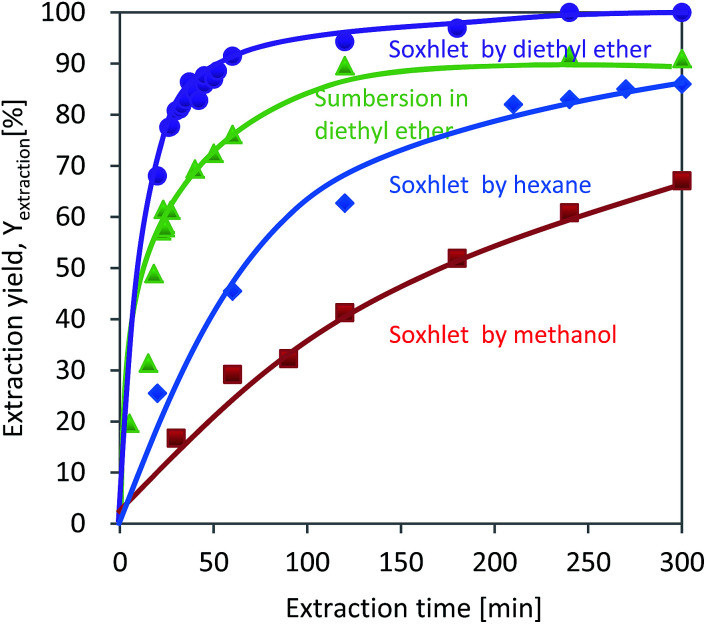
Extraction yields as a function of extraction time and solvent type between 0 and 300 min.

The relationships between *Y*_extraction_ and the extraction time for diethyl ether, methanol, and hexane are summarized in Fig. 2. For all the solvents, *Y*_extraction_ increased with the extraction time. Perfect extraction was achieved by Soxhlet extraction using diethyl ether for 300 min. And extraction by simple submersion in diethyl ether resulted in a lower *Y*_extraction_ than that obtained by Soxhlet extraction. *Y*_extraction_ obtained by submerging reached 77% after 60 min and changed only slowly as a result of further prolonging the extraction time. The *Y*_extraction_ values obtained for methanol and hexane after 300 min, on the other hand, were 67 and 85%, respectively, much lower than that obtained with diethyl ether. Even when the extraction time was extended to 4500 min, methanol and hexane resulted in *Y*_extraction_ values of 82 and 87%, respectively (data not shown).

The lowest and slowest *Y*_extraction_, obtained with methanol, was likely due to the *δ*_SP_ of 14.5 being highly different from that of 8.5 for DINP (Table 1). For diethyl ether and hexane, even though their *δ*_SP_ values are almost the same at 7.4 and 7.3, respectively, the extraction results differed significantly. The different results may be explained by the differences in HSP (*δ*_d_, *δ*_p_, and *δ*_h_). The HSP components of DINP (*δ*_d_ = 16.6, *δ*_p_ = 6.6, and *δ*_h_ = 2.9) differ greatly from those of hexane (*δ*_d_ = 14.9, *δ*_p_ = 0.0, and *δ*_h_ = 0.0), but are similar in value to those of diethyl ether (*δ*_d_ = 14.5, *δ*_p_ = 2.9, and *δ*_h_ = 4.6). From the above results, it can be concluded that the best extraction solvent in the present work was diethyl ether, which achieved complete DINP extraction from the cable samples in 300 min.

### 3.3 Characterization of plasticizer-extracted PVC cables

**Fig. 3:**
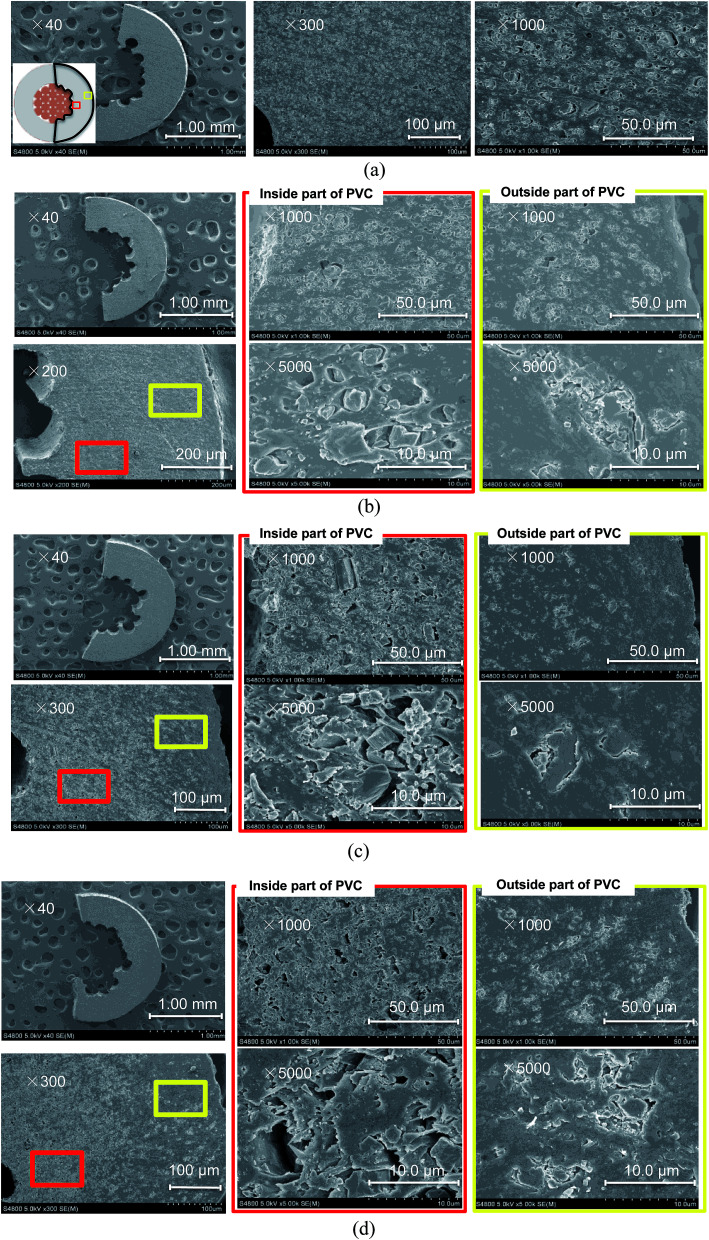
SEM images of cross-sections of PVC covering: (a) original PVC; (b) PVC with 20% DINP extracted; (c) PVC with 60% DINP extracted; and (d) PVC with all DINP extracted. DINP extracted using diethyl ether solvent.

**Fig. 4:**
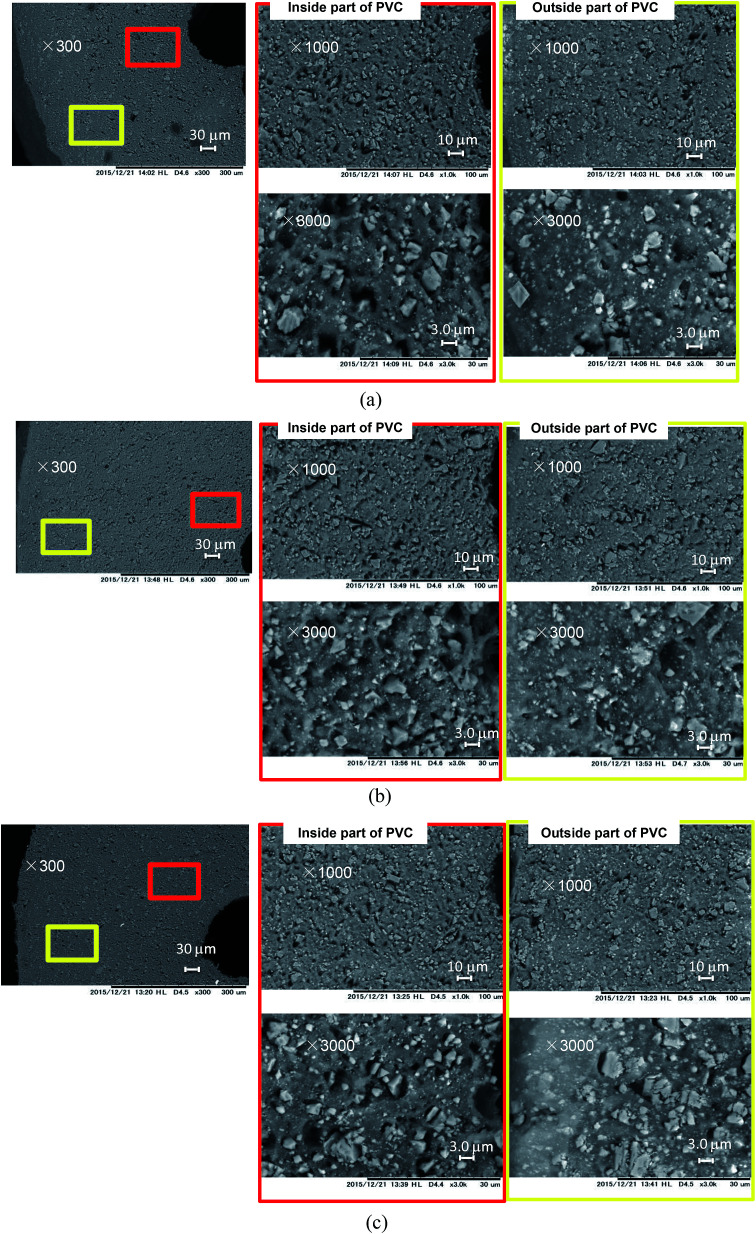
SEM images of cross-sections of PVC: (a) PVC with 59% DINP extracted using diethyl ether; (b) PVC with 59% DINP extracted using methanol; and (c) PVC with 61% DINP extracted using hexane.

**Fig. 5:**
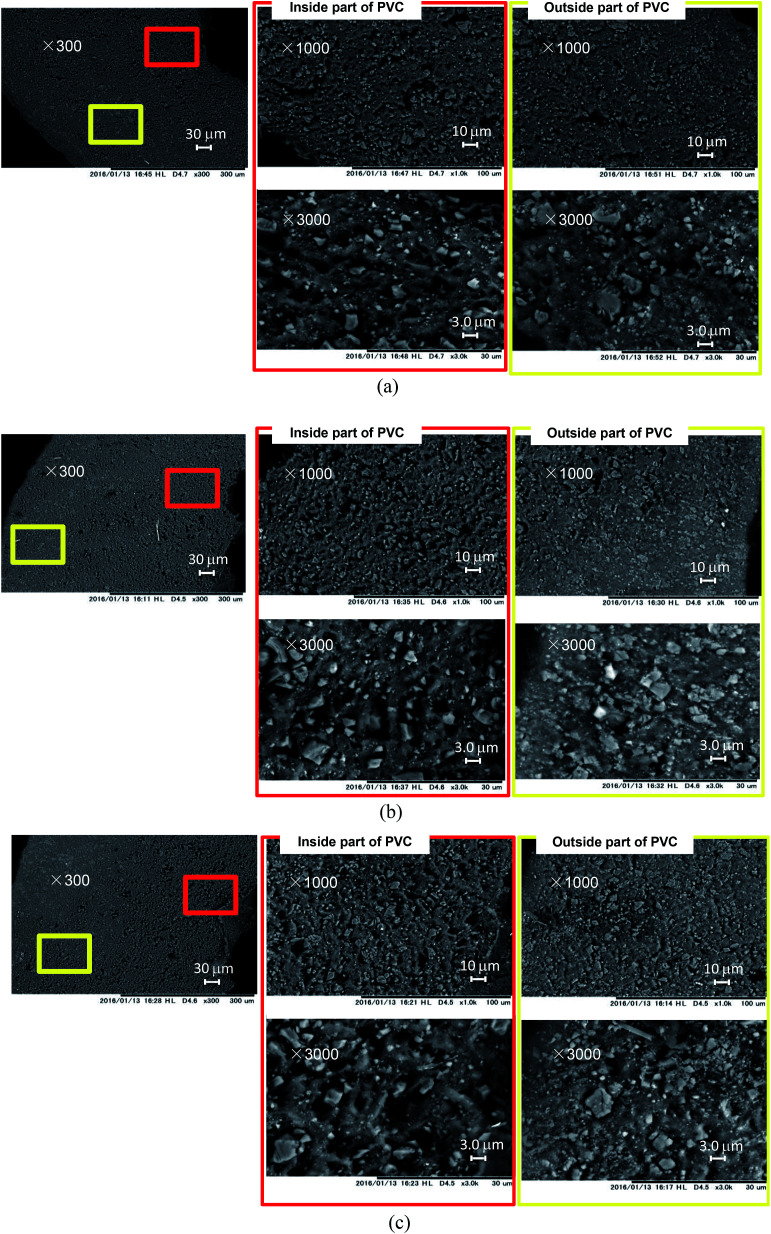
SEM images of cross-sections of PVC: (a) PVC with 83% DINP extracted using diethyl ether; (b) PVC with 82% DINP extracted using methanol; and (c) PVC with 84% DINP extracted using hexane.

SEM images of the cable cross-sections before and after different yields of DINP extraction with diethyl ether are shown in Fig. 3. All the images taken were of half cross-sections of the PVC covering of the electric cables, and the general internal and external viewpoints are marked in the bottom-left inset of Fig. 3(a). In addition, the exact viewpoints of the groups of pictures in Fig. 3(b)–(d) are marked and distinguished by red and yellow blocks for the internal parts of PVC close to the copper wiring and the outside parts close to the cable surface, respectively. The original PVC and that for which 20% DINP had been extracted both had homogeneously distributed pores without significant differences (Fig. 3(a) and (b)). For the samples for which 60 and 100% DINP had been extracted, on the other hand, the morphologies of the internal and external parts were very different (Fig. 3(c) and (d)); the pores in the internal part of the PVC increased in number, while those in the external part decreased. To investigate the effects of the solvent type on the morphology of the PVC covering during DINP extraction, SEM images of the PVC samples for which around 60% of the DINP had been extracted by diethyl ether, methanol, and hexane are shown in Fig. 4(a), (b), and (c), respectively. Likewise, SEM images of the PVC samples for which around 80% of the DINP had been extracted are shown in Fig. 5. No differences can be observed between samples treated by the different solvents for both the 60 or 80% DINP extraction, although the pores observed in those samples for which around 80% of the DINP had been extracted were deeper than those in those samples for which around 60% of the DINP had been extracted. Fig. 4 and 5 showed that the pores could be more clearly identified in the internal parts than in the external parts for a higher *Y*_extraction_ value, which is consistent with Fig. 3. In general, plasticizer is introduced into the PVC matrix by physical kneading. The plasticizer preferentially fills pores and cracks in the PVC matrix that originate during the physical kneading process or from volume shrinkage during the suspension polymerization of the PVC,^40,41^ which results in flexibility.^42^ Based on this, it can be concluded that the pores observed in the SEM images were the result of DINP extraction. The higher concentration of pores in the inner parts of the PVC covering might be due to the greater solvent permeability. Although we do not have concrete evidence to prove this hypothesis, the inner surface of the PVC covering had a ragged surface as opposed to the outer smooth surface (Fig. 3) due to the thin copper wires (each 200 μm in diameter), which may have allowed enhanced solvent permeation.

We also found that the thickness of the PVC covering was reduced after DINP extraction, and that the degree of shrinkage increased with an increase in *Y*_extraction_, as shown in Table 2. The incorporation of plasticizer can make the PVC flexible, which can mechanistically be explained by three theories.^43^ Among these, the free volume theory, a further extension of the lubricity theory by Kirkpatrick^44^ and the gel theory by Aiken *et al.*^45^ and Cadogan *et al.*,^46^ defines free volume as the internal space of a polymer available to polymer chain movements. Plasticizers incorporated into the PVC matrix would increase and maintain the free volume to prevent interactions between neighboring polymer chains, which results in flexibility.^47^ Thus, when the plasticizers are removed, the free volume of the PVC would be expected to decrease, resulting in shrinkage of the PVC covering.

**Table 2:** Shrinking rate (*R*_shrink_) values at different *Y*_extraction_ values using three different solvents

Solvent	*Y*_extraction_ [%]	*L*_0_ [cm]	*L* [cm]	*R*_shrink_ [%]
Diethyl ether	95.7	20.0	18.1	9.6
	85.6	20.0	18.4	7.9
	58.3	20.0	18.9	5.6
	27.4	20.0	19.3	3.5
	12.2	20.0	19.5	2.3
Methanol	62.1	20.0	18.6	7.1
	23.3	20.0	19.4	3.2
	12.1	20.0	19.6	2.0
	6.7	20.0	19.8	1.0
Hexane	70.3	20.0	18.8	6.0
	64.2	20.0	18.9	5.5
	22.9	20.0	19.4	2.8

**Fig. 6:**
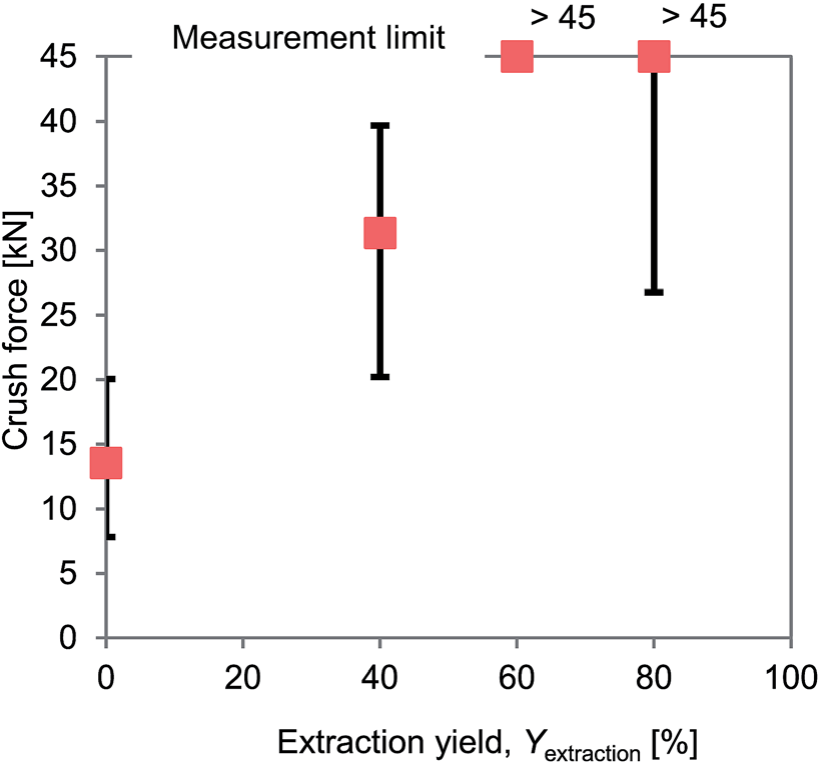
Results of crush resistance tests of PVC samples with different plasticizer extraction yields.

Crush resistance tests were carried out to measure the hardness values of the PVC covering samples for which the DINP had been extracted. The results are summarized in Fig. 6. The crush force increased with *Y*_extraction_, exceeding the measurable limit for samples for which *Y*_extraction_ = 60%. Typical properties of vinyl plastic products containing various amounts of plasticizer have previously been reported and are presented in Table 3.^48^ The reported hardness and tensile strength values decreased as the amount of DINP extracted from the products increased, which indicates that rigid products should require higher crush forces. This is consistent with the crush resistance results obtained in the present work (Fig. 6). The flexural stiffness decreases, and the elongation increases with an increase in the plasticizer content (Table 3), which indicates that flexible PVC should be more deformable when forces act on it than rigid PVC. This would then result in flexible PVC being hard to crush even at very high crush forces. The most important property is the brittleness temperature of rigid products at >23 °C, which means that the PVC covering of electric cables from which 100% of the DINP had been extracted would easily be crushed at room temperature. Therefore, the rigid and flexible PVC may be compared to glass and rubber; glass is hard but brittle, while rubber is soft but elastic, and therefore glass is easily crushed but rubber is not. Since plasticizer extraction causes a flexible PVC covering to become hard and brittle, it makes the PVC easier to crush. Thus, as the hardness increases with *Y*_extraction_, the results of crushing under the same ball milling conditions (and thus the same crush force) will be affected. Therefore, the most appropriate *Y*_extraction_ value for plasticizers extracted from electric cables will make the PVC covering brittle and hard.

**Table 3:** Typical properties of vinyl plastic products

Plastic type	Rigid	Semi-rigid	Flexible
DINP [phr^a^]	0	34	50
DIN	0	25	33

**Typical properties**			
Specific gravity, 20/20 °C	1.4	1.26	1.22
Hardness [Shore A], 15 s	—	94	84
Tensile strength [MPa]	>41	31	21
Flexural stiffness at 23 °C [MPa]	>900	69	12
Elongation [%]	<15	225	295
Brittleness temperature [°C]	>23	−16	−32

a Plasticizer level in parts per hundred resin, by weight.

### 3.4 Effects of extraction yield on separation rate and purity of PVC and Cu

**Fig. 7:**
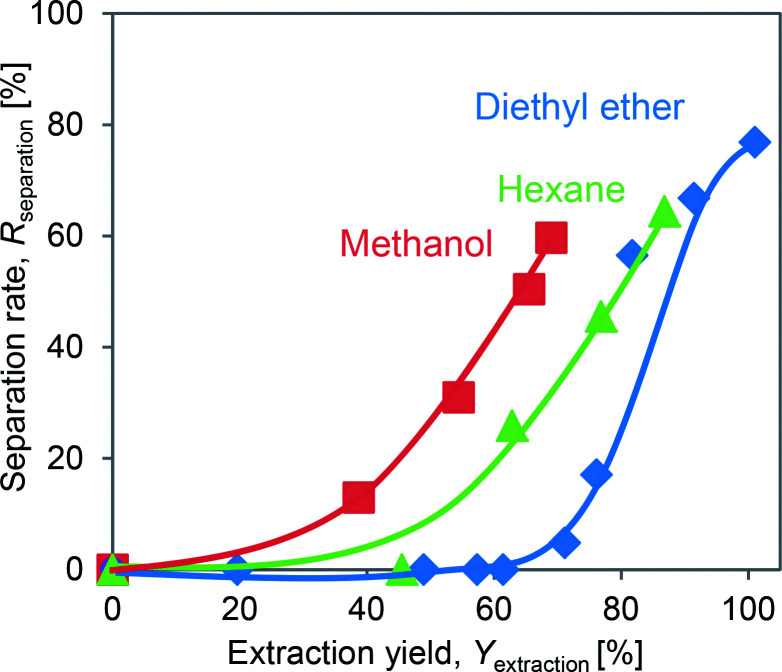
Separation rates obtained at different extraction yields using different solvents.

The effects of the DINP extraction rate and solvent type on the rate of PVC and Cu separation (*R*_separation_) are summarized in Fig. 7. We could not achieve separation when using the original electric cable samples from which no DINP had been extracted. In addition, no separation was observed for those samples extracted by diethyl ether with DINP extraction rates of 20, 49, and 57%, but *R*_separation_ drastically increased to 57% when *Y*_extraction_ reached 82%; for those samples extracted by methanol and hexane, on the other hand, *R*_separation_ increased more gradually with *Y*_extraction_. Furthermore, samples with similar extraction rates of around 60% exhibited very different *R*_separation_ values depending on the type of organic solvent used for the extraction; those samples for which the extraction had been performed using methanol achieved an *R*_separation_ of 50%, while those for which the extraction was done with diethyl ether failed to exhibit any separation. Those for which the extraction was done using hexane achieved an intermediate *R*_separation_ value. Diethyl ether, methanol, and hexane might lead to varying degrees of brittleness of the PVC covering, which could not be detected by SEM analysis; this may then have resulted in the different *R*_separation_ values for the same value of *Y*_extraction_. However, since the use of diethyl ether could result in complete DINP extraction, unlike methanol and hexane, it also resulted in the highest *R*_separation_ value of 77% in the present work.

**Fig. 8:**
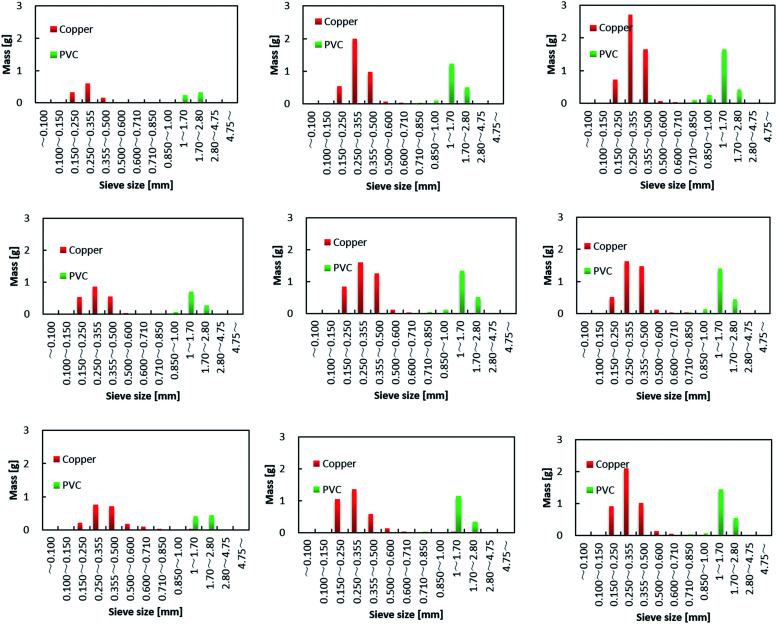
Particle size distributions of crushed samples from which varying amounts of plasticizer had been extracted. Plasticizer extraction yields were: (a) 76, (b) 82, and (c) 100% by diethyl ether; (d) 54, (e) 61, and (f) 69% by methanol; and (g) 63, (h) 77, and (i) 87% by hexane.

**Fig. 9:**
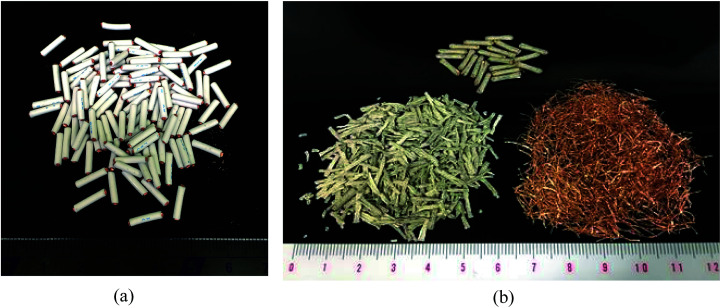
Photos of electric cables: (a) electric cables after plasticizer extraction, and (b) extracted cable samples after ball milling and manual separation.

The separated PVC covering and Cu particle size distributions obtained from samples with different DINP extraction rates are summarized in Fig. 8. The Cu particle size was concentrated in the range <600 μm since the diameter of each Cu wire was around 200 μm. The size of the crushed PVC segments, on the other hand, was concentrated in the range >710 μm. Thus, the separation of roughly crushed PVC covering and Cu wires was very effective, resulting in more than 99% copper purity (*P*_Cu_) regardless of the value of *R*_separation_. Specifically, the maximum *R*_separation_ of 77% (achieved with those samples from which 100% of the DINP had been extracted) also resulted in the highest *P*_Cu_ of >99.8%. Similarly, excellent Cu purity was attained with both methanol- and hexane-treated samples; a Cu purity of >99.8% resulted from samples from which 69% of the DINP had been extracted using methanol and an *R*_separation_ of 60%, as well as from samples from which 87% had been extracted using hexane and an *R*_separation_ of 64%. Hence, a Cu purity of >99% was effectively and easily attained for a size range between 150 and 600 μm. Fig. 9(a) is a photograph of the electric cables after plasticizer extraction by diethyl ether, with the PVC covering shrunken to some degree. Fig. 9(b) shows electric cable samples from which all the plasticizer had been extracted by diethyl ether, followed by manual separation into uncrushed cable samples, recovery of the Cu, and then crushing of the PVC covering. Based on these results, the introduced method of combined plasticizer extraction and ball milling appears to be effective for the recycling of electric cables.

## 4. Conclusions

In summary, we have simultaneously recycled copper and polyvinyl chloride (PVC) from electric cable samples by combining plasticizer extraction and ball milling. In this method, solvents with low boiling points, such as diethyl ether, hexane, and methanol, resulted in good plasticizer extraction efficiency without significant changes to the PVC resin. Among these, diethyl ether could achieve complete diisononyl phthalate extraction from the cable samples, unlike the hexane and methanol. Finally, the maximum separation rate of 77% and highest Cu purity (Cu segments size range: 150–600 μm) of >99% were achieved using samples for which 100% of the plasticizer had been extracted from the PVC resins by diethyl ether, because the PVC became brittle after plasticizer removal. Our work has thus introduced a novel, simple, and effective method for the recovery of both Cu and PVC from electric cables. We believe that the developed approach has large economic advantages because of its low energy costs, inexpensive consumable and recyclable solvents.

## Conflicts of interest

There are no conflicts of interest to declare.

## Supplementary Material

RA-008-C8RA00301G-s001
