# Supplementary material for: Simultaneous recovery of high-purity copper and polyvinyl chloride from thin electric cables by plasticizer extraction and ball milling
Source: RSC Adv. 2018 Feb 13;8(13):6893–903. doi: 10.1039/c8ra00301g (PMC9078293; doi:10.1039/c8ra00301g)
Supplement: RA-008-C8RA00301G-s001 [file RA-008-C8RA00301G-s001.pdf]

Electronic Supplementary Material (ESI) for RSC Advances.
This journal is © The Royal Society of Chemistry 2018

Electronic Supplementary Information (ESI)

Simultaneous recovery of high-purity copper and polyvinyl chloride from thin electric cables by plasticizer extraction and ball milling

Jing Xu, Naoki Tazawa, Shogo Kumagai*, Tomohito Kameda, Yuko Saito and Toshiaki Yoshioka

Graduate School of Environmental Studies, Tohoku University, 6-6-07 Aoba Aramaki-Aza, Aoba-ku, Sendai, Miyagi 980-8579, Japan.

Email: kumagai@env.che.tohoku.ac.jp

Detailed conditions for extraction of diisononyl phthalate

The total amount of extractable plasticizer (diisononyl phthalate [DINP]) from electric cables was determined by Soxhlet extraction using diethyl ether at 75 °C for 24 h.[1] After extraction, samples were dried in vacuum at 40 °C overnight. The weight difference between samples before and after extraction was considered the total amount of plasticizer in the cables.

^{1}H-NMR and ^{13}C-NMR spectra of plasticizer extracted from cable samples

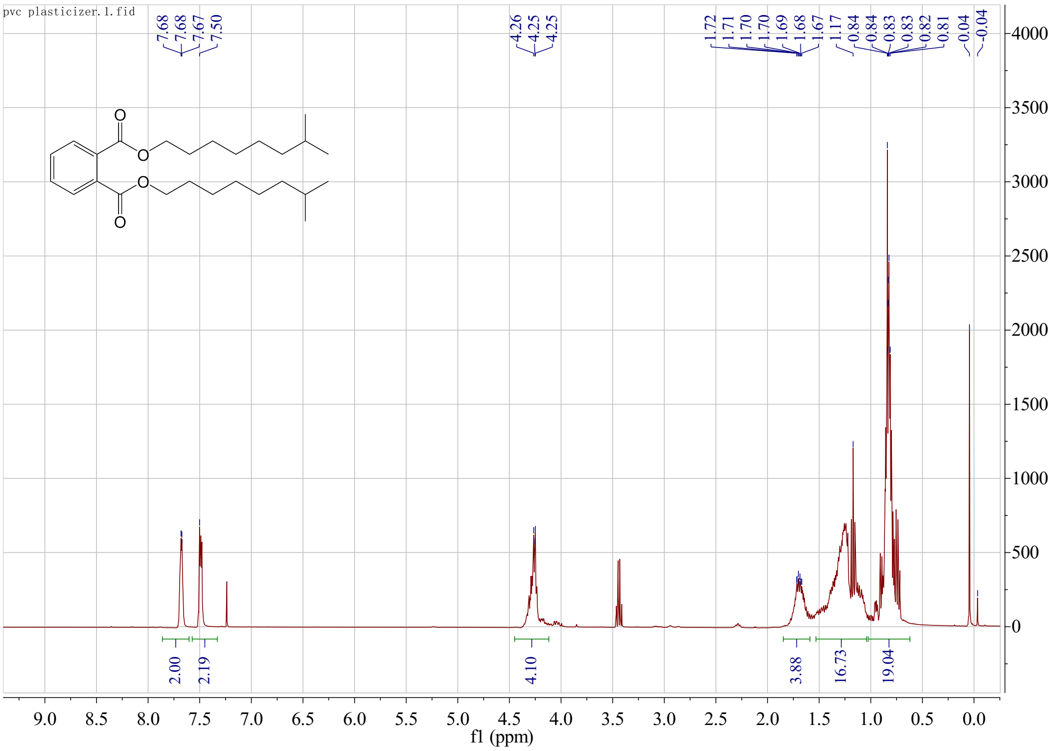

Fig. S1 ^{1}H-NMR of diisononyl phthalate (DINP) extracted from electric cable samples using diethyl ether solvent.

DINP (400 MHz, $CDCl_3$), δ (ppm): 7.68 (2H, H1, dd, 3J1–2 = 5.6 Hz, 4J1–2 = 3.2 Hz), 7.53 (2H, H2, dd, 3J2–1 = 5.6 Hz, 4J2–1 = 3.2 Hz), between 4.44 and 4.40 (around 4H, m), between 2.07 and 0.64 (around 34H, m).

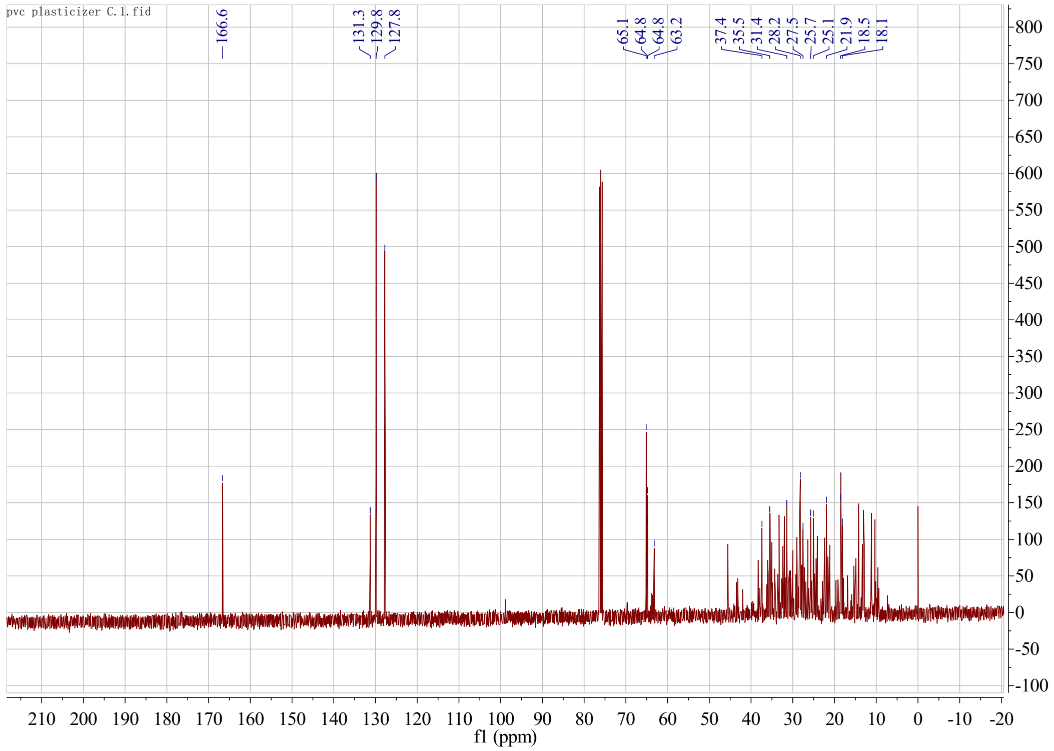

Fig. S2 ^{13}C-NMR of DINP extracted from electric cable samples using diethyl ether.

^{13}C-NMR (400MHz, $CDCl_3$) δ: 166.6, 131.3, 129.8, 127.8, 65.1, 64.8, 63.2, 37.4, 35.5, 31.4, 28.2, 27.5, 25.7, 25.1, 21.9, 18.5, 18.1 .

REFERENCES

1. Fonseca, J. D.; Grause, G.; Kameda, T.; Yoshioka, T., Effects of steam on the thermal dehydrochlorination of poly(vinyl chloride) resin and flexible poly(vinyl chloride) under atmospheric pressure. *Polym Degrad Stabil* **2015,** 117, 8-15.
